# Innovative Optical-Sensing Technology for the Online Fouling Characterization of Silicon Carbide Membranes during the Treatment of Oily Water

**DOI:** 10.3390/s20041161

**Published:** 2020-02-20

**Authors:** Mehrdad Ebrahimi, Axel A. Schmidt, Cagatay Kaplan, Oliver Schmitz, Peter Czermak

**Affiliations:** 1 Institute of Bioprocess Engineering and Pharmaceutical Technology, University of Applied Sciences Mittelhessen, 35390 Giessen, Germany; cagatay.kaplan@lse.thm.de (C.K.); oliver.schmitz@lse.thm.de (O.S.); peter.czermak@lse.thm.de (P.C.); 2 Department R&D, DECKMA Hamburg GmbH, 22525 Hamburg, Germany; axel.a.schmidt@deckma.hamburg; 3 Faculty of Biology and Chemistry, Justus-Liebig University of Giessen, 35390 Giessen, Germany; 4 Fraunhofer Institute for Molecular Biology and Applied Ecology (IME), Project Group Bioresources, 35392 Giessen, Germany

**Keywords:** ceramic membrane, silicon carbide membrane, produced water treatment, oily wastewater, oil-in-water sensor, light scattering

## Abstract

The oil and gas industry generates a large volume of contaminated water (produced water) which must be processed to recover oil before discharge. Here, we evaluated the performance and fouling behavior of commercial ceramic silicon carbide membranes in the treatment of oily wastewaters. In this context, microfiltration and ultrafiltration ceramic membranes were used for the separation of oil during the treatment of tank dewatering produced water and oily model solutions, respectively. We also tested a new online oil-in-water sensor (OMD-32) based on the principle of light scattering for the continuous measurement of oil concentrations in order to optimize the main filtration process parameters that determine membrane performance: the transmembrane pressure and cross-flow velocity. Using the OMD-32 sensor, the oil content of the feed, concentrate and permeate streams was measured continuously and fell within the range 0.0–200 parts per million (ppm) with a resolution of 1.0 ppm. The ceramic membranes achieved an oil-recovery efficiency of up to 98% with less than 1.0 ppm residual oil in the permeate stream, meeting environmental regulations for discharge in most areas.

## 1. Introduction

### 1.1. Produced Water (PW)

Produced water (PW) is a term used in the oil and gas industry to describe the aqueous liquid phase produced from wells during the extraction of oil and gas [1]. PW is the largest source of contaminated water discharged from oil and gas production operations [2] and on a global scale the production rate is 25–39.5 Mm^3^ day^−1^. The composition and physiochemical properties of PW are complex and vary considerably from well to well (Table 1) [3]. The most toxic constituents include oil, salt, sand, heavy metals, bacteria, naturally occurring radioactive materials, and production chemicals. The environmental impact of PW differs according to how it is treated and where it is discharged. Two main approaches are currently recommended for the management of PW: reinjection into the discharged wells, and treatment for reuse followed by beneficial utilization [4,5]. Because oil is the major contaminant in the PW derived from both offshore and onshore drilling operations, its recovery has become the most important environmental aspect of PW treatment.

**Figure 1:**
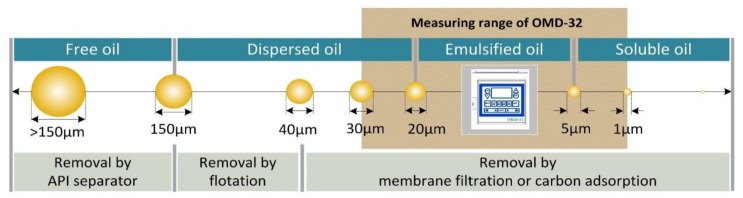
Technologies for the treatment of produced water according to oil droplet size. API = American Petroleum Institute, OMD-32 is an oil-in-water sensor.

The oil fraction in PW is a combination of dissolved oil, emulsified oil, dispersed oil and free oil [6,7,8,9,10,11] depending on the droplet size (Figure 1). Several physical, biological and chemical treatment technologies can be used individually or in combination to recover the oil from these phases [3,4,5,8] including gas flotation, hydrocyclonic separation, decanter separation, phase separation, membrane filtration, and adsorption. Given the diverse properties of PW in terms of the nature and concentration of different oil droplet sizes, as well as the small difference in specific gravity between heavy oil and water, most of these technologies do not achieve the increasingly stringent quality criteria for treated water [12,13,14]. 

The inability of current technologies to process the increasing volumes of PW means that unacceptable levels of oil remain present in discharged or recycled water [13]. Oil and gas producers must, therefore, develop improved technologies for the processing of PW to ensure that the quality of treated water complies with the latest regulations [14].

### 1.2. Ceramic Membrane Technology for PW Treatment

Organic/inorganic membrane separation technology has emerged as a promising alternative for industrial wastewater treatment, particularly for the treatment of PW [15,16]. The advantages of membrane filtration include low energy consumption, a small footprint, no requirement for additional chemicals during the treatment process, and consistent effluent quality [17]. Furthermore, inorganic (ceramic) membranes have remarkable thermal and physiochemical stability under harsh process and feed conditions, such as extreme pH, temperature and pressure, and the presence of organic solvents [18,19]. However, these advantages depend on the type of membrane [20]. Some inorganic membranes are composed of metal oxides such as aluminum oxide (Al_2_O_3_), titanium oxide (TiO_2_), zirconium oxide (ZrO_2_), and silicon oxide (SiO_2_) [6,21]. Others are based on zeolites, microporous carbon, or silicon carbide (SiC). Several ceramic membrane have been evaluated for their microfiltration (MF), ultrafiltration (UF) and nanofiltration (NF) performance during the treatment of PW, particularly membranes based on Al_2_O_3_, TiO_2_ and ZrO_2_ [19,22,23,24,25,26]. 

SiC membranes have received little attention compared to metal oxides, especially for the treatment of PW [26,27,28]. SiC membranes are characterized by a strongly hydrophilic surface, high porosity, and a rather uniform pore size distribution [16,17]. The surface characteristics of the membrane material such as isoelectric point, surface charge density, and hydrophobicity are among the factors that may influence membrane performance. Fouling mechanisms, i.e., cake forming and pore blocking, which cause a permeate flux decline in the filtration process are strongly influenced by the surface charge of the membrane [29]. The degree and intensity of membrane fouling and their cleaning efficiency are dependent on the zeta-potential of the feed suspension particles and of the membrane surface. The retention rate of the membrane depends also on the pH value of the feed and the isoelectric point of the membrane material used. In general, the retention of the membrane is lower near the isoelectric point. The isoelectric point of SiC (2.5–3.5) is significantly lower than that of other ceramic materials, such as TiO_2_ (5.1–6.4), Al_2_O_3_ (8.0–9.4) and ZrO_2_ (6.3–7.1) [30]. This should ensure higher membrane flux during the treatment of PW while limiting the impact of fouling [12]. 

### 1.3. Oil-in-Water Sensors for PW Treatment Applications

The environmental protection agencies of several oil- and gas-producing countries mandate a maximum monthly average oil content of 29 parts per million (ppm) in treated PW [31]. Therefore, oil producers must regularly measure and report oil-in-water concentrations at production facilities to manage their separation and discharge processes. Several techniques can be used to measure oil-in-water concentrations, such as focused ultrasonic acoustics, fiber optic chemical sensing, image analysis, light scattering and turbidity, photoacoustic sensing and ultraviolet (UV) fluorescence, including laser-induced fluorescence [32]. The main problem with many of these methods is their limited accuracy and precision. For example, real-time water quality monitoring is not possible with chemical methods because they require complex offline chemical reactions. We focus on the use of optical sensors, which have advantages over both chemical and physical methods [33] and are currently used in numerous research and commercial applications.

## 2. Objectives

The work presented herein is based on our previous studies focusing on the development of single and multistage processes using different ceramic membrane materials and geometries (the filtration layer is mainly composed of Al_2_O_3_, TiO_2_ or ZrO_2_) for the efficient treatment of tank-dewatering produced water (TDPW) and prepared oily model solutions (OMSs) [22,23,34,35,36,37]. During the treatment of oily wastewater, the continuous measurement of the oil content in permeate samples is a key indicator of the effectiveness of ceramic membranes under different process conditions, revealing the overall performance of the treatment system. An online oil-in-water sensor that achieves the accurate and stable online measurement of oil levels in oily wastewater is a key technology for discharge permit compliance monitoring. Accordingly, this study has two objectives:

- To evaluate the application, performance and fouling behavior of SiC MF and UF membranes for the separation of oil during the treatment of TDPW and prepared OMSs.
- To characterize a novel online oil-in-water sensor based on light scattering for (i) the continuous measurement of oil content during the filtration of oily wastewater samples (permeate, concentrate, and retentate samples) and (ii) the evaluation of process parameters that affect membrane performance, specifically the crossflow velocity (CFV) and transmembrane pressure (TMP).

## 3. Theory of Light Scattering to Determine the Oil-in-Water Content

### 3.1. Overview

Light scattering is already widely used for the online monitoring of bilge water (in the shipping industry), cooling water, surface runoff and PW [38,39,40,41]. It is a highly sensitive and robust method that can easily detect oil contamination at levels as low as 0.1 ppm. The technique measures the light scattered by oil droplets, and determines the oil-in-water content based on the resulting optical properties (elastic scattering). Modern detectors accommodate different types of oil so the instrument does not need to be calibrated according to the nature of the sample (lifetime calibration). However, measurements can be influenced by the presence of solid particles and gas bubbles. The angle of detection must, therefore, be set to detect oil droplets while suppressing non-oil components. In early instruments, the rule of thumb was that oil droplets induced small scatter angles (10–30°) whereas particles and other non-oil components tended to scatter light at wider angles. However, the relationship is more complex than this in reality and it is necessary to look more closely into the underlying theory, which was described by Mie in 1908 [42]. The theory has been applied to many different aspects of light scattering [43,44,45] but here we focus on the interaction between light and droplets/particles in water.

### 3.2. Light-Scattering Theory

Mie distinguishes three different light-scattering regimes according to the particle diameter relative to the wavelength of the incident light in water [42]. If the particle diameter is much smaller than the incident wavelength, the optical regime is known as Rayleigh scattering. For particles of approximately the same size as the incident wavelength, the optical regime is Mie scattering. Finally, for particles that are much larger than the incident wavelength, light scattering follows the Fraunhofer diffraction regime, in which most of the light is scattered forward in a small cone and variations in the optical properties of the scattering particles generate only small variations in the scatter pattern. 

**Figure 2:**
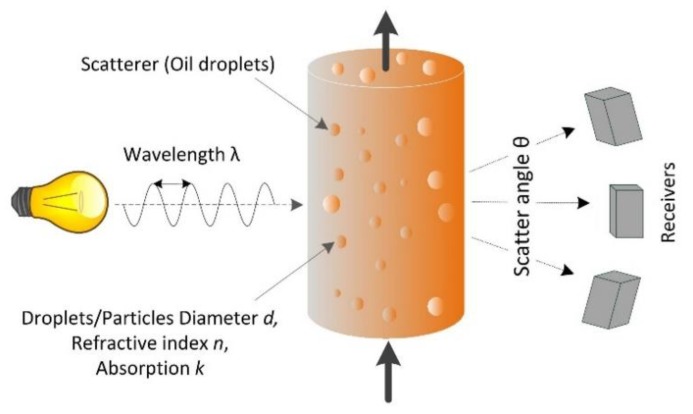
Principles of light scattering.

The principles of the light-scattering method are summarized in Figure 2. A narrow-band light source such as a laser diode emits light at a specific wavelength that interacts with the particles or oil droplets. The scatter pattern depends on the incident wavelength, the particle/droplet diameter, and its optical properties (the refractive index relative to the matrix, absorption coefficient, and shape). The scattered light intensity is measured as an angular distribution. Information gathering is inherently limited to objects that interact with light, and characteristics that are related to optical properties. Therefore, scattered light measurements are insensitive to constituents that are completely dissolved, and measurement is not affected by differences in the gravitational density of the particle or oil droplet. Scattered light instruments, therefore, measure the oil content in ppm by volume rather than mg·L^−1^.

### 3.3. Typical Light-Scatter Pattern

**Figure 3:**
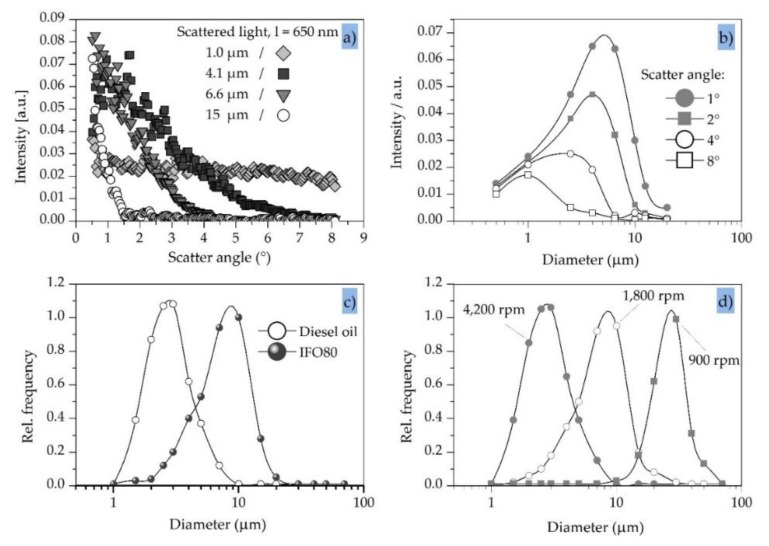
Characteristics of scattered light detection using model particles/droplets. (**a**) Typical scatter pattern using monodisperse particles made of polystyrene/IF080 (sticky crude oil). (**b**) Sensitivity of specific scatter angles to different particle diameters (λ = 650 nm). (**c**) Droplet size distribution using a three-stage rotational pump at different revolution speeds. (**d**) Droplet size distribution in diesel oil using a three-stage rotational pump for two different oil types.

Typical light-scatter patterns were generated by illuminating suspensions of monodisperse polystyrene particles with an active surface coating to prevent agglomeration, thus mimicking the shape and optical properties of oil droplets. We plotted the relative scattered light intensity against the scatter angle for particles ranging in diameter from 1.0 to 15 µm, and overlaid the calculated values (Figure 3a). All samples were prepared with a constant volume of polystyrene. A 650 nm laser diode was used as the light source combined with a complementary metal oxide semiconductor (CMOS) linear image sensor (512 pixels, 25 µm pitch) as a light receiver array. 

The measured values aligned well with the calculated values, and the experiment confirmed that the bigger the particle the smaller the cone of scattered light. For example, most of the light scattered by the 15-µm particles fell within a ~1° scatter angle. This would place a high demand on the optics and electronics in a light-scattering detector, but the scatter angle can be increased by using a longer incident wavelength and increases proportionately. Accordingly, a 980 nm near infrared laser would increase the scatter angle by a factor of 1.5, albeit at the expense of the light-scattering intensity (which would fall to ~20% of the value achieved using the 650 nm laser). This might, nevertheless, improve the measurement. Wavelengths >1050 nm are typically excluded because this approaches the performance limits of silicon-based light receivers and the absorption coefficient of water also increases significantly.

### 3.4. Light-Scatter Sensitivity Versus Particle Diameter

The sensitivity of particular scatter angles to different particle sizes is shown in Figure 3b. For example, the scattered light intensity at 4° is similar for the 1.0 µm and 4.1 µm particles but negligible for the 6.6 µm and 15 µm particles, indicating that the measurement of light scattered at 4° achieves adequate sensitivity, but only for particles with a diameter up to ~4.0 µm. Figure 3b compares the sensitivity of 1°, 2°, 4° and 8° scatter angles for an incident wavelength of 650 nm. Clearly, the smaller the scatter angle the greater the range and variation in sensitivity. For example, at the lowest scatter angle of 1° the sensitivity to 6.0 µm particles is three times higher than the sensitivity to 1.0 µm and 12 µm particles. Therefore, if a broad and flat band of sensitivity is needed to detect particles that vary in size, a single scatter angle will be insufficient. A combination of different scatter angles is necessary to generate the required sensitivity profile. Moreover, the ability to design a sensitivity curve provides the opportunity to build devices that can switch between a broad and flat sensitivity band and the ability to discriminate between particles with different sizes.

### 3.5. Light Scattering and Oil Droplet Diameter

Given the flexibility of light-scattering detectors in terms of particle diameter, oil-detection systems should match the size range of droplets in specific applications [46,47,48,49]. This will depend in part on the pump used to bring water into the measurement device and also the nature of the valves and other passive flow components, which typically reduce the average diameter of oil droplets due to shear forces. The measurement of two types of oil under similar conditions is shown in Figure 3c. The average particle size of the crude sticky oil (IFO80) is approximately three times greater than the light raffinate diesel oil, and the comparison indicates that typical sensors will need to detect droplets in the size range 1.0–30 µm. The typical droplet size distribution of the diesel oil after mixing with a three-stage rotational pump at 50 °C is shown in Figure 3d. As expected, the average droplet size is reduced at higher pump speeds, reflecting the greater energy input and higher shear forces. The average diameter changes approximately tenfold (3.0–30 µm) when comparing the highest pump speed (4200 rpm) to the lowest (900 rpm). 

## 4. Materials and Methods

### 4.1. Oil-in-Water Sensor Specifications

**Figure 4:**
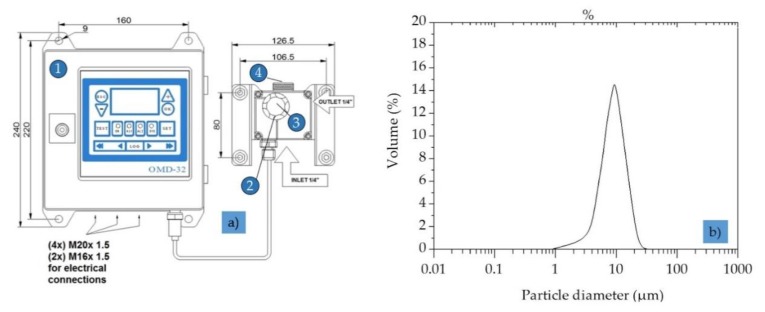
Oil-in-water sensor to measure the particle size distribution. (**a**) Oil-in-water sensor OMD-32: 1 = computer; 2 = measuring cell; 3 = desiccator cap; 4 = head screw; (**b**) particle size distribution in the prepared OMS.

The online oil-in-water sensor OMD-32 (Table 2, Figure 4a), developed and manufactured by DECKMA Hamburg GmbH (Hamburg, Germany), was designed specifically for use in oil–water separator units. 

The specification and performance of the oil-in-water sensor exceeds the requirements of the International Maritime Organization (IMO) for 15 ppm oil-in-water monitors as set out in Resolution MEPC.107. An optical sensor array measures a combination of light scattered and absorbed by oil droplets in the sample stream. The sensor signals are then sent to a microprocessor to produce a linear output. If an alarm is triggered (set point = 10 ppm), the two oil alarm relays are activated following an adjusted time delay. The microprocessor continuously monitors the condition of the sensor components and associated electronics to ensure that calibration accuracy is maintained over time and during exposure to extreme environmental conditions. 

### 4.2. Oily Wastewater Specifications

To ensure standardized conditions during the filtration experiments, we prepared OMSs differing in dispersed oil content and droplet size distribution. The OMSs were prepared by the pre-emulsification of crude oil (Bramberge oilfield, North Sea, Germany) with a rotor stator homogenizer, followed by single-pass processing with an Emulsiflex C5 high-pressure homogenizer (Avestin Europe GmbH, Mannheim, Germany) at 450 bar. 

The final concentration of dispersed oil was adjusted to 30–35 ppm by dilution with demineralized water. The droplet size distribution of the feed solution (Figure 4b) was measured by light scattering (Mastersizer S, Malvern Instruments, Malvern, UK) with an accuracy of ±2% on Volume Median Diameter. Samples of TDPW were obtained from German BP AG, Emsland Oil Refinery, Lingen, Germany (Table 3).

### 4.3. Ceramic Membrane Specifications

The characteristics of the SiC MF and UF membranes used in this study are shown in in Table 4. 

### 4.4. Filtration Unit Specifications

**Figure 5:**
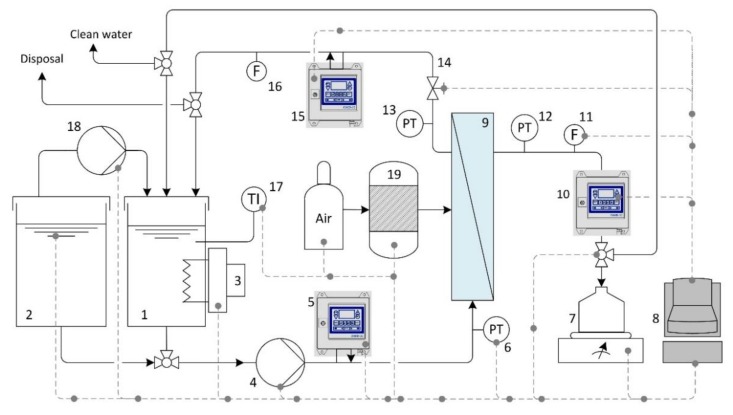
Schematic representation of the semi-automatic crossflow filtration unit: (1 + 2) feed tanks; (3) heading element; (4 + 18) pumps; (5 + 10 + 15) oil-in-water sensors; (6 + 12 + 13) pressure transducers; (7) balance; (8) automation and process control system; (9) ceramic membrane module; (11 + 16 + 20) flow meters; (14) valve; (17) temperature transducer; (19) back flush tank.

All experiments were carried out using the filtration pilot plant shown in Figure 5 with different settings for the CFV (0.25–7.1 m·s^−1^) and TMP (0.5–3.0 bar). The filtrations were conducted in fed-batch mode or total recycle mode to keep the concentration in the feed tank constant.

The transmembrane pressure (TMP) for a typical membrane module determines the driving force of the pressure-driven membrane processes and is calculated as shown in Equation (1):

(1) $$\mathrm{TMP}=\frac{P_{feed}+P_{retentate}}{2}-P_{filtrate}$$

where $P_{feed}\;$= the inlet pressure on the feed side (bar),$\;P_{retentate}$ = the outlet pressure on the retentate side (bar), and $P_{filtrate}$ = the pressure on the filtrate side (bar).

At the beginning of each filtration experiment, the SiC membranes were tested with deionized water at different TMPs (0.5, 1.0, 1.5 and 2.0 bar) and a constant CFV of 1.0 m·s^−1^ to determine the pure water permeability and membrane resistance using Darcy’s formula [50,51,52] as shown in Equation (2):

(2) $$J=\frac{\mathrm{TMP}}{µ\cdot R}$$

where J = membrane permeability to pure water (L·h^−1^·m^−2^), R= resistance to mass transfer associated with the clean membrane, and µ = the dynamic viscosity of the solution (Pa·s).

Membrane separation is characterized mainly in terms of permeate flux and rejection efficiency. At any time during the filtration, the total resistance of a fouled membrane ($R_{t})\;$is the sum of the intrinsic membrane resistance and the membrane fouling resistance. $R_{t}\;$can be described using the resistance-in-series model if the feed contains components other than water [27] as shown in Equation (3):

(3) $$R_{t}=R_{m}+R_{f}$$

where $R_{t}\;$= total membrane resistance (m^−1^),$\;R_{m}\;$= intrinsic membrane resistance (m^−1^), and $R_{f}$ = time-dependent membrane fouling resistance (m^−1^).

$R_{f}$ can be apportioned to hydraulically reversible fouling resistance ($R_{rev}$) and irreversible fouling resistance ($R_{irrev}$). $R_{rev}$ is caused by the formation of a cake layer or by the concentration polarization of materials on the membrane surface. It can be limited if the CFV is high and may be removed by chemical cleaning. $R_{irrev}\;$is caused by the persistent adsorption of pollutants (dissolved and colloidal material) on the membrane surface or in the entrance to membrane pores. $R_{irrev}$ cannot be eliminated by either moderate physical or chemical cleaning [51,52,53,54]. $R_{f}$ is calculated as shown in Equation (4) [51,55]:

(4) $$R_{f}=R_{rev}+R_{irrev}$$

where $R_{rev}\;$= resistance of reversible fouling (m^−1^) and $R_{irrev}$ = resistance of irreversible fouling (m^−1^). 

Combining Equations (3) and (4) yields Equation (5):

(5) $$R_{t}=R_{m}+(R_{rev}+R_{irrev})$$

The membrane-fouling resistance ($R_{f})\;$was determined by measuring the pure water flux of the membrane after filtration and after rinsing the membrane with pure water to remove any residual particles from the surface. We then subtracted the resistance of the clean membrane [56]. Equation (3) can be expanded as shown in Equation (6):

(6) $$R_{f}=\;R_{t}-R_{m}\;=\frac{\mathrm{TMP}}{µ\cdot J_{f}}-R_{m}$$

where$\;J_{f}\;$= the water flux of fouled membrane, before chemical cleaning (L·h^−1^·m^−2^).

All filtration streams (permeate, retentate and concentrate) were fed through the online sensor to measure and record the oil concentration continuously with a time resolution of 1 s. The online sensors were installed in a bypass and connected to the SCADA system (Labbox, HiTec Zang GmbH, Herzogenrath, Germany). The oil rejection efficiency$\;(R_{oil}$) was calculated using Equation (7) [9,27]:

(7) $$R_{oil}=\frac{C_{f}-C_{p}}{C_{f}}\cdot \;100$$

where$\;R_{oil}$ = oil rejection efficiency (%),$\;C_{f}$ = the oil concentration in feed samples (mg·L^−1^), and$\;C_{p}=\;$the oil concentration in permeate samples (mg·L^−1^). 

The percent adsorption/deposition of the oil in the filtration tests was calculated using Equation (8) [27]:

(8) $$adsorption/deposition=\frac{C_{t0}\cdot V_{t0}-\;C_{tn}\cdot V_{ftn}}{C_{t0}\cdot V_{ft0}}\cdot 100$$

where$\;C_{t0}$ = the initial oil concentration in the feed tank (mg·L^−1^),$\;C_{tn}$ = the oil concentration in the feed tank at time t_n_ (mg·L^−1^), $V_{ft0}$ = the initial volume of feed at time t_0_ (L), and $V_{ftn}\;$= the volume of feed at time t_n_ (L).

### 4.5. Standard Chemical Cleaning Procedures

After each filtration experiment, the membranes were chemically cleaned with a 1% solution of P3 Ultrasil-14 (Henkel AG and Co. KGaA, Herborn, Germany). The cleaning solution was circulated in the equipment for 2 h at 60 °C. The equipment was emptied and the membrane was rinsed with demineralized water. After cleaning, the pure water permeability of the membrane was measured and the flux recovery efficiency was calculated using Equation (9).

(9) $$\mathrm{FRE}=\frac{J_{WC}}{J_{W0}}\cdot \;100$$

where FRE = the flux recovery efficiency (%),$\;J_{WC}\;$= the water flux after chemically cleaning (L·h^−1^·m^−2^), and$\;J_{W0\;}$= the initial water flux of virgin (unused) membrane (L·h^−1^·m^−2^).

## 5. Results and Discussion

### 5.1. Characterization of Sensor Performance—Effects of Oil Concentraion and Temperature

The signal response and linearity of the output signal of the oil-in-water sensor were tested with TDPW and OMSs prepared by diluting stock solutions of a defined droplet size with demineralized water (Figure 4b). The oil content of the test solutions was measured with intermittent flushing of the measuring cell using clean water. Each concentration was measured for 30 s and the values were averaged. The data were processed by linear regression and the standard error and coefficients of determination were calculated. 

**Figure 6:**
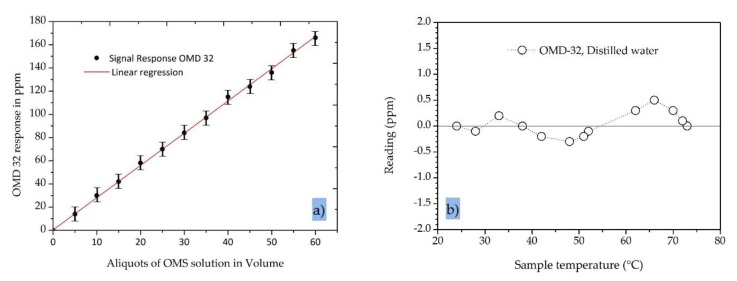
Properties of the OMD-32 online oil-in-water sensor. (**a**) The signal response of the sensor when tested with an oily model solution (OMS). (**b**) The temperature dependence of the sensor in distilled water.

The results recorded by the OMD-32 sensor with the modified/extended measurement range are shown in Figure 6a. In all experiments, we observed a strong linear correlation between the oil concentration and the sensor signal output (R^2^ = 0.999). The standard error was ±1.9 ppm, indicating that the device can accurately determine the residual oil contamination in the range 0–200 ppm. The standard error of 0.6 ppm for the OMD-32 sensor is far better than the 5.0 ppm specified in the IMO regulations for 15 ppm bilge alarms.

To determine the temperature dependence of the OMD-32 sensor, the device was mounted in a closed loop with a heater and a pump in series and was fed with distilled water while increasing the temperature. We observed a slight variation in the signal (less than ±0.5 ppm) over the temperature range 20–80 °C, which is again far better than the 5.0 ppm specified in the IMO regulations for 15 ppm bilge alarms (Figure 6b).

### 5.2. Characterization of Membrane Performance—Effects of Operating Parameters

Membrane flux is influenced by factors such as physiochemical feed composition/properties, membrane-specific characteristics, and hydrodynamic conditions, thus affecting the capability of the membrane to remove oil during the filtration of oily wastewaters. Our previous investigation of PW treatment with different ceramic MF, UF and NF membranes [22,23,34,35,36,37] revealed that membrane fouling is a major challenge, causing a significant decline in permeate flux and in some cases reducing oil retention efficiency. Membrane fouling phenomena must therefore be understood in order to control membrane-based filtration processes [57,58,59]. TMP and CFV are critical operational parameters for MF and UF membranes, so we investigated the effect of different TMPs and CFVs on membrane fouling.

### 5.3. Effect of Crossflow Velocity (CFV) and Transmembrane Pressure (TMP)

**Figure 7:**
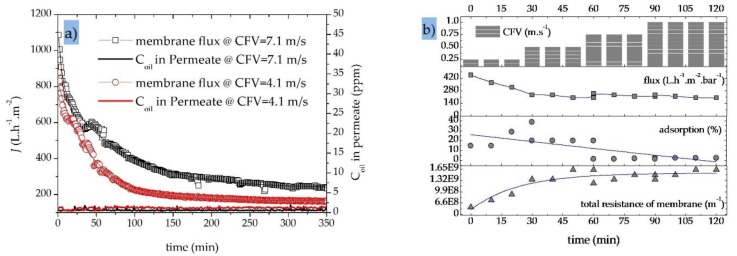
Variations in the permeate flux under different conditions. (**a**) Variations in permeate flux with time for a SiC UF membrane (40 nm) at different crossflow velocities (CFVs) (4.1 and 7.1 m·s^−1^) and the corresponding oil content in permeate samples; transmembrane pressure (TMP) = 2.0 bar; fed-batch mode; OMS initial oil concentration = 30 ppm; T = 50 °C. (**b**) Variations in permeate flux, oil adsorption/desorption and total residence of the membrane with time for a SiC MF membrane (200 nm); CFV = 0.25, 0.5, 0.75 and 1.0 m·s^−1^; initial oil concentration = 200 ppm; total recycle mode; T = 40 °C, TMP = 1 bar.

Figure 7a shows the decline in permeate flux and the resulting permeate oil concentrations for a 40 nm UF membrane during the treatment of an OMS with an initial oil concentration of 30 ppm. We tested the effect of two different CFVs (4.1 and 7.1 m·s^−1^) at a constant TMP of 2.0 bar in fed-batch mode and observed different trends in the decline of permeation flux and oil rejection during the first 150 min. This probably reflects the initial formation of a variable fouling layer composed of oil droplets on the membrane surface [55]. During the first 150 min, the permeate flux declined by 75% (from 878 to 223 L·h^−1^·m^−2^) at a CFV of 4.1 m·s^−1^ and by 65% (from 1085 to 390 L·h^−1^·m^−2^) at a CFV of 7.1 m·s^−1^. Subsequently, the flux declined at a slow and steady rate in both experiments. If the permeation flux trends are considered for the total duration of the filtration experiment rather than a limited running time, the CFV shows an even more significant positive influence on membrane performance. The average membrane permeate flux over the entire duration of the filtration experiment (350 min) was 361 L·h^−1^·m^−2^ for a CFV of 7.1 m·s^−1^, which is ~50% higher than the 244 L·h^−1^·m^−2^ observed at a CFV of 4.1 m·s^−1^ [60].

Figure 7a also shows that during the treatment of OMS, increasing the CFV reduces the probability of oil particles accumulating on the membrane surface, reducing the degree of fouling and achieving a higher quasi-steady-state permeate flux (165 L·h^−1^·m^−2^ at a CFV of 4.1 m·s^−1^ compared to 240 L·h^−1^·m^−2^ at a CFV of 7.1 m·s^−1^ after a filtration time of 250 min) [15]. At a constant TMP of 2.0 bar, the higher CFV increases the performance of the SiC UF membrane without limiting its oil retention capacity over the entire duration of the filtration experiment (~6 h). The UF membrane therefore achieves an excellent oil removal efficiency of >98% at both CFVs (Table 5).

Figure 7b shows the effect of CFV on the permeate flux through a 200 nm SiC MF membrane in total recycle mode, as well as oil adsorption on the membrane surface/pores and total membrane resistance. At a constant TMP of 1.0 bar, increasing the CFV from 0.25 to 1.0 m·s^−1^ causes more shear stress at the membrane surface during the first 30 min of the filtration run, reducing the average rate of adsorption/deposition of oil particles from 23% to <7%. This in turn results in a quasi-constant trend of total membrane resistance (1.63·10^9^ m^−1^) and an average membrane flux of 221 L·h^−1^·m^−2^ by increasing the CFV to >0.5 m·s^−1^. 

**Figure 8:**
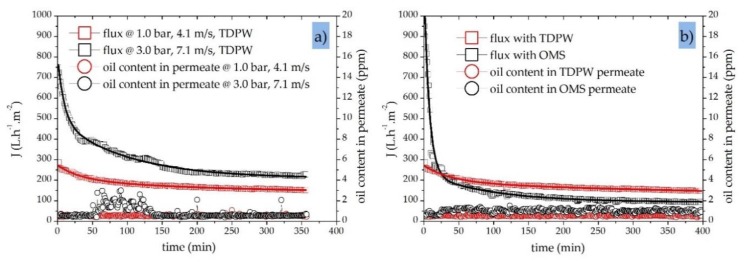
Permeate flux and oil content in permeate samples for a 40 nm SiC UF membrane. (**a**) Permeate flux and oil content during the filtration of TDPW at different TMPs (1.0 and 3.0 bar) and different CFVs (4.1 and 7.1 m·s^−1^); T = 50 °C in fed-batch mode; initial oil concentration in feed = 200 ppm. (**b**) Permeate flux and oil content during the filtration of TDPW (initial oil concentration = 200 ppm) and OMS (initial oil concentration = 30 ppm); TMP = 1.0 bar; CFV = 4.1 m·s^−1^; T = 50 °C in fed-batch mode.

Next, we investigated the influence of CFV and TMP on the performance of a 40 nm SiC UF membrane while changing the feed composition and characteristics in filtration experiments with both OMS and TDPW. Figure 8a shows the typical decline in permeate flux through the membrane and the associated oil concentration in the permeate stream over time during the treatment of TDPW using two combinations of TMP and CFV (1.0 bar/4.1 m·s^−1^ and 3.0 bar/7.1 m·s^−1^). Under the lower TMP/CFV conditions, the initial membrane flux was 270 L·h^−1^·m^−2^, whereas under the higher TMP/CFV conditions it was ~2.7-fold higher at 750 L·h^−1^·m^−2^, indicating that a fouling layer accumulates during the first hour of filtration. Accordingly, the decline in membrane flux during the first hour was significantly steeper (~50%) when the higher TMP/CFV conditions were applied, compared to ~12% under the lower CFV/TMP conditions. 

The online oil-in-water sensor recorded an average residual permeate oil concentration <0.6 ppm during the filtration of TDPW at a TMP of 1.0 bar and a CFV of 4.1 m·s^−1^. However, in experiments with the TMP increased to 3.0 bar, the oil retention capacity showed a different trend, and a higher oil content was observed in the permeate samples. The oil content in the permeate stream at a TMP of 3.0 bar increased from an average of 0.6 ppm to 3.8 ppm at times, but stayed constant at 0.6 ppm when the TMP was lower. This indicates that, under the higher TMP/CFV conditions, the critical TMP and CFV values have already been exceeded with regard to the oil retention capacity of the membrane. 

The deformation of oil droplets occurs at higher TMPs, increasing the probability that oil droplets will pass through the membrane. Numerical simulations have been carried out to investigate the phenomenon of oil droplet deformation and breakup, and the critical pressure of permeation for oil droplets trapped in membrane pores with a circular cross-section [60]. During the membrane-based treatment of oily wastewater, oil droplets can deform and pass through membrane pores if the TMP and CFV exceed the critical pressure and critical crossflow shear, respectively [61]. Accordingly, oil droplets blocking the membrane pores can penetrate the membrane if the TMP is high enough. A similar phenomenon appears to be responsible for the higher oil concentrations found in the permeate samples in our experiments.

Figure 8b shows the transient permeate flux behavior of a 40 nm SiC UF membrane and the resulting oil concentration in permeate samples during the filtration of TDPW and OMS at a TMP of 1.0 bar and a CFV of 4.1 m·s^−1^. The initial flux during the treatment of OMS was 1000 L·h^−1^·m^−2^, which is four times higher than the 270 L·h^−1^·m^−2^ recorded during the treatment of TDPW. Oil is the only fouling component in the OMS, whereas the complex makeup of TDPW (Table 3) provides additional contaminants that cause immediate membrane fouling. This immediate fouling layer generates an additional barrier, thus the average oil content in the TDPW samples (initial C_oil_ = 200 ppm) was 0.5 ppm, compared to 1.0 ppm for the OMS samples (initial C_oil_ = 30 ppm). However, the total separation efficiency in both cases exceeded 98% (Table 5). 

**Figure 9:**
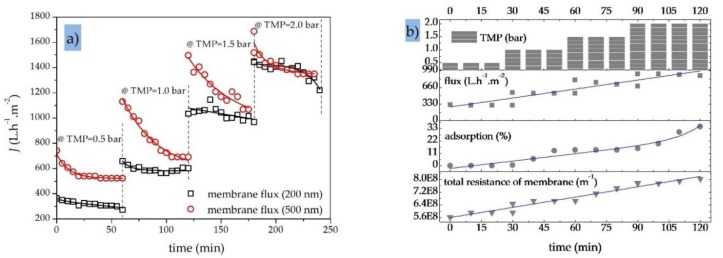
Performance of SiC MF membranes. (**a**) Membrane fluxes for two SiC MF membranes (200 and 500 nm) at varying TMPs (0.5, 1.0, 1.5 and 2.0 bar); CFV = 1.0 m·s^−1^; OMS initial oil concentration = 100 ppm; T = 40 °C; total recycle mode. (**b**) Variations in permeate flux, oil adsorption and total membrane residence for a SiC MF membrane (200 nm) at varying TMPs (0.5, 1.0, 1.5 and 2.0 bar); CFV = 1.0 m·s^−1^; initial oil concentration in feed = 200 ppm; T = 40 °C; total recycle mode.

Figure 9a shows the effect of gradually increasing the TMP (from 0.5 to 2.0 bar) on the permeation flux of two SiC MF membranes with molecular weight cut-off values of 200 and 500 nm. The total recycle mode experiments with an OMS (initial C_oil_ = 100 ppm) were carried out at a constant CFV of 1.0 m·s^−1^ and a process temperature of 40 °C. Initially, the flux through both MF membranes increased with the increase in TMP, consistent with earlier reports concerning the treatment of oily wastewater with ceramic MF membranes [10,62]. However, a decrease in permeate flux was observed over the duration of the filtration (1 h) at both TMPs, which is the main challenge limiting the practical applications of MF in oily wastewater treatment [61]. The decrease in membrane flux may vary depending on the process and feed conditions, membrane material, and pore size. Figure 9a also indicates that the higher TMP of 0.5 bar caused fouling to occur more rapidly during each filtration run while significantly reducing the permeation rate of the fouled membrane. However, the increase in permeate flux at TMPs >1.0 bar was not linear, in agreement with an earlier study [62]. Higher TMPs cause oil droplets to pass through the membrane pores, thus more oil droplets accumulate on the membrane surface and in the pores, leading to a higher fouling rate [63]. Membrane fouling was more severe at the larger pore size of 500 nm (Figure 9a). 

When an OMS with an initial oil concentration of 100–200 ppm was processed using a 200 nm SiC MF membrane, increasing the TMP from 0.5 to 2.0 bar caused a significant increase in the initial permeate flux but also in the amount of oil adsorption on the membrane, which rose from 0 to 35% (Figure 9b). This also led to a quasi-linear increase in the total membrane resistance from 5.6·10^8^ to 8.0·10^8^ m^−1^ [63]. At higher TMPs, more severe fouling was observed because oil and particulates that pass through the membrane are adsorbed and accumulate within the pore channels [25,61].

### 5.4. Membrane Chemical-Cleaning Procedure

Membrane fouling is classed as reversible if it can be controlled by physical or chemical cleaning and irreversible if it accumulates over time and cleaning cannot restore the original membrane performance [64]. The main foulants are organic, colloidal and mineral, which account for 50%, 30% and 20%, of fouling respectively [58]. The purpose of chemical cleaning is to reverse the loss of membrane permeability and restore flux and rejection by removing the foulants described above [54,59]. 

To specify the flux recovery efficiency (FRE) of the SiC membranes, we measured the pure water permeability of virgin (unused) membranes and fouled membranes before and after chemical cleaning. We then used linear regression to plot the pure water flux (*J*) against the TMP in the range 0.5–2.0 bar. To determine whether TMP is a relevant parameter affecting membrane cleaning and the resulting membrane regeneration efficiency, we carried out MF/UF experiments with OMS under a range of TMP values between 0.5 and 3.0 bar (Table 5). 

**Figure 10:**
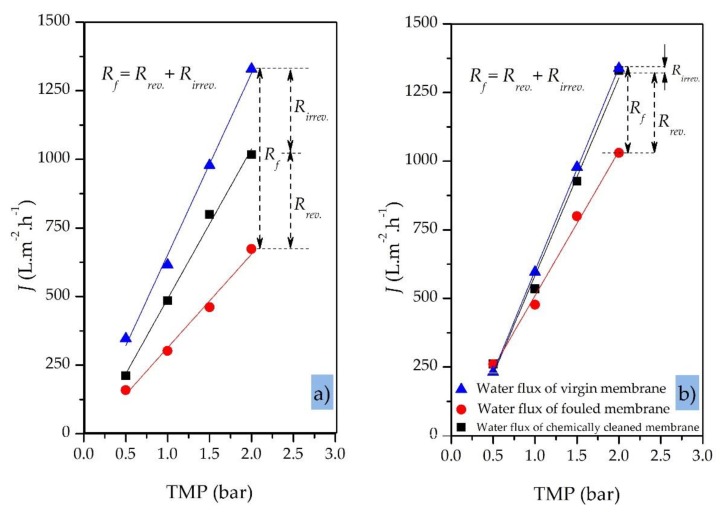
Pure water flux for a 200 nm SiC MF membrane (virgin, fouled and cleaned) against transmembrane pressure. (**a**) Variable TMP (0.5–2.0 bar); CFV = 1.0 m·s^−1^; C_oil_ of oily model solution = 100 ppm; T = 40 °C; total recycle mode. (**b**) Constant TMP = 1.0 bar; other parameters as above.

Figure 10a,b compare the clean water fluxes (plotted against TMP) for a virgin 200 nm SiC MF membrane before the filtration run, the same membrane after the treatment of OMS (fouled membrane), and the same membrane after chemical cleaning. The membrane was fouled in total recycling mode under a gradually increasing TMP (from 0.5 to 2.0 bar) while the CFV and temperature were kept constant at 1.0 m·s^−1^ and 40 °C, respectively. The initial pure water flux of the virgin SiC membrane was 347 L·h^−1^·m^−2^ at 0.5 bar and 1329 L·h^−1^·m^−2^ at 2.0 bar. 

Our results showed that the higher the applied TMP (>2.0 bar) during the filtration of oily wastewaters, the greater the degree of irreversible fouling of the membrane and thus the lower the efficiency of chemical cleaning at the end of the filtration process (Figure 10a). When the TMP was >2.0 bar, irreversible fouling of the membrane could not be eliminated completely using standard cleaning procedures based on NaOH alone, regenerating only 80% of the original membrane flux. An additional citric acid cleaning step was needed to increase the regeneration efficiency to >90%. In contrast, a moderate and constant TMP of ≤1.0 bar resulted in a membrane cleaning efficiency of >98% using standard cleaning procedures based on NaOH alone (Figure 10b, Table 5). At TMPs ≤1.0 bar, the limited fouling layer can be removed from the membrane surface and pores by standard chemical cleaning to restore the initial pure water flux. 

## 6. Conclusions

We investigated a membrane-based process using tubular SiC ceramic MF and UF membranes (500, 200 and 40 nm) in crossflow mode for the treatment of oily wastewater. The TMP and CFV were varied and the process performance was investigated in terms of permeate flux (fouling phenomena) and permeate quality (oil rejection efficiency). We also tested a novel online oil-in-water sensor based on the principle of light scattering.

Our study confirms the suitability of OMD series monitors for the supervision and control of PW treatment processes by cross-flow filtration and shows that scattered light measurement is an accurate, reliable, rapid and effective optimization tool for oily wastewater treatment processes. The online oil-in-water sensor achieved continuous measurement of the oil content in the OMS and TDPW samples. The standard error of the online sensor was ±1.9 ppm, indicating the suitability of this device for the accurate determination of residual oil contamination in industrial applications where the detection range is 0–200 ppm. Moreover, we found that the OMD-32 device was robust across a wide temperature range (20–80 °C) showing less than ±0.5 ppm variation in readings. This is far better than the 5.0 ppm threshold stipulated by IMO regulations for 15 ppm bilge alarms.

We found that increasing the TMP initially achieved an increase in permeate flux but also promoted the adsorption of oil particles on the MF and UF membrane surface and, thus, increased the total membrane resistance. At TMPs > 2.0 bar, more severe fouling was observed because oil droplets and particulates that pass through the membrane are more likely to be adsorbed, and thus accumulate within the membrane pores. Increasing the CFV generally improved the permeate flux in both the MF and UF systems. The permeate quality (residual oil contamination) appeared to be unaffected by the TMP in the range 0.5–2.0 bar and CFV in the range 0.25–7.1 m·s^−1^, resulting in the removal of up to 98% of the oil leaving permeate residues of less than 1.0 ppm, easily meeting and exceeding regulatory limits for discharge in most areas. Increasing the oil content of the concentrate stream from 30 to 200 ppm had no significant influence on the efficiency of oil removal and the 5.0 ppm alarm threshold for the oil-in-water sensor was never triggered.

Finally, we found that standard chemical cleaning procedures were effective for the SiC ceramic membranes as long as the TMP is maintained at <2.0 bar. Under these conditions, the fouling layer does not appear to be pronounced, and the foulants can be removed from the membrane surface and pores by standard cleaning using NaOH to restore the initial pure water flux.

## Figures and Tables

**Table 1:** Main contaminants found in produced water and their concentrations. TSS = total suspended solids; TDS = total dissolved solids; TOC = total organic carbon.

Major Parameter	Value	Unit
TSS	<1000	mg·L^−1^
TDS	<400,000	mg·L^−1^
TOC	0–1500	mg·L^−1^
Total organic acids	<10,000	mg·L^−1^
Total oil	2–600	mg·L^−1^
pH	4.3–10	–
Density	1014–1140	kg·m^−3^
Oil droplet size	2.0–30	μm

**Table 2:** Specifications of the oil-in-water online sensor (OMD-32).

Range	0–200 ppm
Accuracy	up to ±1.0 ppm below 10 ppm
Resolution	1.0 ppm (0.1 ppm below 10 ppm)
Response time	<5.0 s
Sample water pressure	max. 10 bar
Sample water temperature	up to 90 °C

**Table 3:** Characteristics of oily model solutions (OMSs) and tank-dewatering produced water (TDPW) compared to oilfield produced water (PW). Conductivity data for the oilfield PW were not available (n.a.).

Major Parameter	OMS	TDPW	Oilfield PW
pH	7.0–7.8	4.3–10	4.0–10
TOC	20–100 mg·L^−1^	200–2000 mg·L^−1^	0–1500 mg·L^−1^
Dispersed oil	30–300 mg·L^−1^	200–1000 mg·L^−1^	2.0–400 mg·L^−1^
Conductivity	160–220 µS·cm^−1^	20,000–80,000 µS·cm^−1^	n.a.

**Table 4:** Characteristics of the SiC microfiltration (MF) and ultrafiltration (UF) membranes. Membrane manufacturer: 1 = Saint-Gobain Industrie Keramik Roedelheim GmbH, Roedental, Germany; 2 = LiqTech International A/S, Ballerup, Denmark.

Parameter	Description		
Nominal pore size	500 nm	200 nm	40 nm
pH range	0–14	0–14	0–14
Membrane length	0.45 m	0.45 m	0.25 m
Number of channels	37	37	31
Channel diameter	0.0034 m	0.0034 m	0.0030 m
Membrane filtration area	0.18 m^2^	0.18 m^2^	0.09 m^2^
Manufacturer	1	1	2

**Table 5:** Selected flux and oil retention data for SiC MF and UF membranes.

Cut-Off (nm)	Feed Source	Filtration Mode	CFV (m·s^−1^)	TMP (Bar)	Duration (min)	Temp. (°C)	C_Oil_ Feed (ppm)	Initial Flux L·h^−1^·m^−2^)	Final Flux (L·h^−1^·m^−2^)	Flux Decline (%)	FRE (%)	$R_{oil}\;(\%)$
40	OMS	FBM	4.1	1.0	420	50	30	950	88	91	99	>98
40	OMS	FBM	4.1	2.0	420	50	30	878	162	82	95	>98
40	OMS	FBM	7.1	3.0	420	50	30	1086	235	78	80	>97
40	TDPW	FBM	4.1	1.0	420	50	200	275	148	46	90	>98
40	TDPW	FBM	7.1	3.0	400	50	200	1163	229	80	79	>96
200	OMS	FBM	1.0	1.0	240	40	100	777	600	23	99	>98
200	OMS	TRM	1.0	1.0	240	40	200	590	455	23	97	>98
200	OMS	TRM	1.0	0.5	240	40	200	573	506	12	98	>96
200	OMS	TRM	1.0	0.5	240	40	100	363	273	25	97	>98
500	OMS	TRM	1.0	1.0	240	40	200	2260	317	84	94	>98

FBM: fed-batch mode; TRM: total recycle mode; FRE: flux recovery efficiency; OMS = oily model solution; TDPW = tank dewatering produced water.

## Abbreviations

The following abbreviations are used in this manuscript:

**API**	**American petroleum institute**	**OMS**	**oily model solution**
Al_2_O_3_	aluminum oxide	Ppm	parts per million
$C_{f}$	initial oil concentration in feed	PW	produced water
$C_{t0}$	initial oil concentration in feed tank	$P_{feed}$	pressure on the feed side
$C_{tn}$	oil concentration in feed at the time $t_{n}$	$P_{retentate}$	pressure on the retentate side
C_oil_	oil concentration	$P_{filtrate}$	pressure on the filtrate side
$C_{p}$	oil concentration in permeate	$R_{t}$	total membrane resistance
CFV	cross flow velocity	$R_{f}$	membrane fouling resistance
CMOS	complementary metal oxide semiconductor	$R_{m}$	membrane resistance
COD	chemical oxygen demand	$R_{oil}$	oil rejection efficiency
FRE	flux recovery efficiency	SiC	silicon carbide
$J_{f}$	water flux of fouled membrane	SiO_2_	silicon oxide
$J_{W0}$	pure water flux of fresh membrane	TDS	total dissolved solids
$J_{Wc}$	pure water flux after cleaning	TDPW	tank dewatering produced water
kDa	Kilodalton	TiO_2_	titanium oxide
$L_{w}$	membrane permeability	TOC	total organic carbon
MF	Microfiltration	TMP	transmembrane pressure
MWCO	molecular weight cut-off	TSS	total suspended solids
µ	dynamic viscosity	UF	ultrafiltration
NF	Nanofiltraion	$V_{ft0}$	initial volume of feed at the time $t_{0}$
		$V_{ftn}$	initial volume of feed at the time $t_{n}$
